# Influence of Wavelength on Light Transmittance, Heat Generation and Pulpal Cellular Response Across Different Restorative Procedures

**DOI:** 10.4317/jced.63873

**Published:** 2026-02-26

**Authors:** Sharanya Singh, Mateus Garcia Rocha, Alexandre Silvino, Shannon M. Wallet, Mario Alexandre Coelho Sinhoreti, Dayane Oliveira

**Affiliations:** 1 Department of Restorative Dental Sciences, College of Dentistry, University of Florida, 1395 Center Drive, 32610, Gainesville, FL, USA; 2 Institute of Macromolecules, Federal University of Rio de Janeiro, 2030 Horacio Macedo Ave, 21941-598, Rio de Janeiro, RJ, Brazil; 3 Department of Oral Biology, College of Dentistry, University of Florida, 1395 Center Drive, 32610, Gainesville, FL, USA4; 4 Department of Restorative Dentistry, Piracicaba Dental School, State University of Campinas, 901 Limeira Ave, Areiao, 13414-903, Piracicaba, SP, Brazil

## Abstract

**Background:**

This study aimed to evaluate the effects of different wavelengths on light transmittance, heat generation, and pulpal cellular response across different restorative procedures.

**Material and Methods:**

Standardized 5mmx5mm Class I preparations and dentin discs were prepared, leaving 0.5mm of dentin at the pulpal floor using extracted third molars. Direct restorations on the Class I preparations were performed using conventional or bulk-fill composites, and 1.5mm ceramic restorations were cemented onto the dentin discs with resin cement. Light-curing procedures were performed using blue (460nm), green (520nm), and red (620nm) wavelengths at 1000 mW/cm². The degree of conversion was assessed using FTIR-ATR (Nicolet iS20). Specimens were positioned above the input sensor of a spectrophotometer (MARC-LC) for the light-transmittance analyses or in a customized oral cavity chamber simulator for temperature variations analyses using an infrared thermal camera (FLIR ONE PRO), with measurements performed on the pulpal floor. Odontoblast-like cells (MDPC-23) were exposed to the same maximum radiant exposure that reached the pulp chamber, and cell viability was assessed using an MTT assay. A power analysis was conducted to determine the sample size to provide a power of at least 0.8 with =0.05. Statistical analyses were performed using one-way ANOVA and Tukey's test.

**Results:**

Degree of conversion did not differ across wavelengths/irradiance conditions (p&gt;0.09). Across all restorative procedures tested, light transmittance to the pulpal floor increased with longer wavelengths (p&lt;0.05). Increased material thickness resulted in significant reductions in transmittance across all wavelengths (p&lt;0.001), with longer wavelengths less affected by this attenuation. Thermal analysis demonstrated that shorter wavelengths produced significantly greater temperature increases within the pulp chamber (p&lt;0.05), while red light consistently produced the lowest changes (p&lt;0.01). Cell viability analysis revealed that red light maintained cell viability at levels statistically similar to those of non-irradiated controls (p=0.23); blue and green light significantly reduced cell viability (p&lt;0.05).

**Conclusions:**

Within the limitations of this in vitro model, it was possible to conclude that longer wavelengths demonstrate increased light transmission, lower temperature rise, and higher cell viability compared to shorter wavelengths delivered at equal exposure.

## Introduction

Dental practitioners commonly use various light-cured materials nowadays, including resin-based composites for direct restorations and resin-based cements for cementing indirect restorations (1). Resin-based materials contain monomers, such as dimethacrylates, which polymerize to form a solid matrix (1). The polymerization process converts carbon-carbon double bonds (C=C) into single C-C bonds1 , and it is initiated by photoinitiators activated by specific wavelengths (1). The most common photoinitiator used nowadays is camphorquinone, with an absorption spectrum ranging from 360 to 510nm and a maximum absorption peak at 468nm (2). Accordingly, many curing lights emit predominantly blue light, with a spectrum ranging from 420 to 500nm and a peak at 460nm. However, multiwave light-curing units have been available for more than two decades and incorporate additional violet wavelengths (380-420nm) to activate alternative photoinitiators, such as 2,4,6-trimethylbenzoyl-diphenylphosphine oxide (TPO) and bisacylphosphine oxide (BAPO) (3). TPO has an absorption spectrum range from 380-425nm and a maximum absorbance peak of 400nm; and BAPO has an absorbance spectrum range from 365-416 nm and a maximum absorbance peak of 400 nm (2). The inclusion of different wavelengths in multiwave curing lights resulted in a nonuniform light beam, regardless of the type of emission tip used to compensate (4). Light transmission between 380 and 420nm tends to decrease with depth, affecting the depth of cure of composites that contain photoinitiators that absorb light in this spectrum range (4). This can have clinical implications for composites containing Camphorquinone, as the nonuniform spectral emittance distribution, also known as the beam profile, of the curing light can affect polymerization homogeneity, particularly when photoinitiators differ in their absorption characteristics (5). Multiwave curing lights exhibit considerable variations in radiant and spectral emittance between the center and the periphery of the emission tip due to the spatial arrangement of the different-wavelength-emitting LED chips (5). As a result, the position of the curing light can affect polymerization outcomes, different areas of the material are exposed to varying levels of spectral and radiant intensity (5). Beyond material considerations, interest has grown regarding biological responses to light exposure. Shorter wavelengths, such as violet and blue lights, are highly energetic and carry/deliver more photon energy than other visible wavelengths at similar radiant emittances (6 - 8). Photon energy not absorbed by the photoinitiators in the resin-based material is released as heat into the surrounding tissues (7 , 9). In vitro studies suggest that heat generated by these wavelengths can damage cells by disrupting metabolic processes and compromising protein stability, thus impairing cellular function and reducing viability. Thermal stress denatures proteins, alters enzyme activity, and compromises membrane integrity, thereby contributing to cellular damage (9 , 10). Blue light toxicity is not limited to heat generation; it can also induce metabolic breakdown, potentially leading to adverse effects on cellular health (10). Although the clinical relevance of temperature rise during light curing remains debated, laboratory studies continue to investigate strategies that may minimize pulpal exposure, particularly in conditions with thin remaining dentin. In contrast, longer wavelengths exhibit opposite characteristics, allowing for greater light transmittance and increased depth of cure in resin-based materials (6 , 8). Moreover, longer wavelengths carry less photon energy than shorter wavelengths, thus associated with reduced heat generation and, potentially, a lower risk of damage to pulp and gingival tissues (11). However, there is currently limited information on how different single-wavelength light sources interact with tooth structure and restorative materials regarding transmission, heat generation, and pulpal cellular response. Therefore, there is a compelling rationale for exploring the potential of long wavelengths in restorative dentistry. For this reason, this in vitro study aimed to evaluate the effects of different monochromatic wavelengths (blue, green, and red) on light transmittance, heat generation, and pulpal response in various restorative procedures, including direct and indirect restorations. The tested hypotheses of this study were: (H1) Long wavelengths would result in greater light transmittance through different restorative materials compared to shorter wavelengths; (H2) Long wavelengths would generate less heat and induce less cell damage compared to short wavelengths.

## Material and Methods

- Resin-based materials formulation Table 1 lists the monomers and filler particles used in the experimental resin-based materials.

Table 1

**Figure 1:**
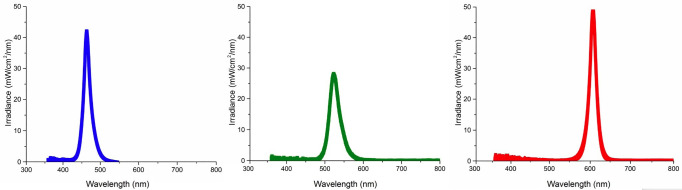
Spectral emittance (mW/cm2/nm) for each tested LED against wavelength (nm).

Different resin-based dental materials were mechanically blended (SpeedMixer, DAC 150.1 FVZ-K, Hauschild Engineering, Hamm, North Rhine-Westphalia, Germany). The adhesive system consisted of 10 wt% urethane dimethylmethacrylate (UDMA), 10 wt% triethylene glycol dimethacrylate (TEGDMA), 25 wt% (hydroxymethyl)methacrylate (HEMA), and 55 wt% ethanol (Sigma Aldrich, St. Louis, MO, USA). The resin cement consisted of 20wt% bisphenol A-glycidyl methacrylate (BisGMA), 10wt% UDMA, and 10wt% TEGDMA loaded with 60wt% of filler particles, in which, 10wt% consisted of 50 nm fumed silica and 50wt% of 0.7 m BaBSiO2 (Esstech Inc., Essington, PA, USA). The conventional composite consisted of 10wt% BisGMA, 11.5wt% Bisphenol A ethoxylate dimethacrylate (BisEMA), 11.5wt% UDMA, and 2wt% TEGDMA loaded with 65wt% of filler particles, in which, 13wt% consisted of 50 nm fumed silica and 52wt% of 0.7 m BaBSiO2. The bulk fill composite consisted of 16wt% BisEMA, 6wt% Exothane 24 (Esstech Inc., Essington, PA, USA), and 1wt% TEGDMA loaded with 77wt% of filler particles, in which, 2wt% consisted of 16 nm fumed silica and 75wt% of 7.5 m BaBSiO2. A coordination complex12 was added to all formulations in 0.2wt% as a multi-wavelength absorption photoinitiator combined with 2wt% of diphenyliodonium hexafluorophosphate (IOD) and 3wt% of ethyl-4-(dimethylamino)benzoate (EDMAB) (Sigma Aldrich, St. Louis, MO, USA). For all materials formulated, first, the monomers were mechanically blended. Then, the photoinitiator and its additives were added to the resin blend. For the loaded resin materials, the filler particles were added subsequently, first by pre-mixing the fumed silica with the resin blend for 30 seconds at 3000rpm, followed by the BaBSiO2 filler for 1 minute at 3500rpm. Then, mixed one final time for 1 minute at 3500rpm. Class I preparation for the direct restorative procedures One hundred and twenty extracted third molars were prepared to simulate Class I preparations for direct restoration with conventional or bulk fill composites (n=10). Extracted human third molars were obtained as discarded clinical specimens under approval of the University of Florida Institutional Review Board (IRB202201548). Class I preparations were standardized with 5 mm long, 5 mm wide, and 4 mm deep, leaving 0.5 mm of dentin at the pulpal floor. First, the occlusal surface of each tooth was flattened using a polishing machine (AUTOMET 250, Buehler, Lake Bluff, IL, USA). Then, the roots from the teeth were sectioned to expose the pulp floor to acquire an intaglio view of the pulp, so that the final pulp thickness could be measured with a dental caliper. The Class I preparations were prepared with a cylindrical diamond dental bur in a rotary hand drill (Kavo Dental, Charlotte, NC, USA) coupled to a cavity preparation machine (Odeme Dental Research, Pompano Beach, FL, USA) with three axes and dimensional control accuracy of 0.01 mm. For standardization purposes, the light-transmittance through the Class I preparations was evaluated to randomize the samples. - Dentin and ceramic discs preparation for the indirect restorative procedures Dentin discs with 5x5 mm were obtained from sixty extracted third molars to simulate the tooth substrate for the indirect restorations to be cemented with resin cement (n=10). First, the roots from the teeth were sectioned to expose the pulp floor of each tooth, then made flat using a polishing machine (AUTOMET 250, Buehler, Lake Bluff, IL, USA). Then, a 0.5mm dentin slice of the pulpal floor from each tooth was obtained using a precision cutting machine (Isomet 1000, Buehler, Lake Bluff, IL, USA) with a diamond blade 15 LC (dimensions: 102 mm in diameter, 0.3 mm in thickness, Buehler, Lake Bluff, IL, USA) under water cooling at 400 rpm. The indirect restorations were prepared using CADCAM lithium disilicate ceramic blocks (IPS Emax, Ivoclar Vivadent, Schaan, Liechtenstein), shade A1, high translucency. 1.5mm slices of the ceramic blocks were sectioned using the precision cutting machine under the same settings previously described. The ceramic discs were fired in the Ivoclar Vivadent Programat furnace according to the manufacturer's instructions. The final thickness accuracy of all discs was verified using a digital caliper (Mitutoyo Corp., Kawasaki, Japan) with a precision of ± 0.1 mm. Light transmittance through all discs was used to standardize the randomization of the samples. - LEDs Characterization LEDs emitting different wavelength spectra (blue (460nm), green (520nm), and red (620nm)) (Chanzon, Shenzhen, China) were coupled to a power supply (Tekpower, Montclair, CA, USA) capable of controlling their voltage (V) and amperage (A). Light from each LED was delivered through a fiber-optic light guide (9 mm in diameter), and voltage and current were set for each LED to achieve a radiant emittance of 1000 mW/cm2. The spectral characteristics, including mean irradiance (mW/cm²) according to wavelength range (nm), were characterized for each LED using a calibrated spectrometer (MARC Resin Calibrator, BlueLight Analytics, Halifax, Canada) (Fig. 1).

1

All light-curing procedures were performed with the guide held perpendicular to the specimen surface and in contact with the specimen (0 mm tip-to-specimen distance). - Resin-based formulation pre-testing The degree of conversion (DC) of the experimental resin composites was assessed using Fourier Transform Infrared Spectroscopy (FTIR) coupled with an attenuated total reflectance (ATR) accessory (Nicolet iS20, Thermo Fisher Scientific, Waltham, MA). Specimens (n = 5) were light-cured for 10 seconds. The FTIR spectra of both the unpolymerized and polymerized samples were recorded, with 16 scans performed at a resolution of 1 cm-¹. The degree of conversion was calculated by comparing the ratio of absorbance of aliphatic C=C bonds (1638 cm-¹) to that of aromatic C=C bonds (1608 cm-¹) in both polymerized and unpolymerized states using the following formula:

(1) $$\mathrm{DC}(\%)=(1-\frac{\frac{X_{a}}{Y_{a}}}{\frac{X_{b}}{Y_{b}}})\times 100$$

Where, Xa and Xb are the absorbance of the aliphatic double bonds in the polymerized and unpolymerized states, respectively. Ya and Yb are the absorbance of the aromatic double bonds in the polymerized and unpolymerized states, respectively. All resin-based materials tested demonstrated similar degree of conversion rates regardless of wavelength exposure (blue (460nm), green (520nm), or red (620nm)), when light cured for 10s (p=0.0935) or 20s (p=0.176). - Light Transmittance Analysis The light transmittance through the different restorative procedures was measured with a spectrophotometer (MARC Resin Calibrator, BlueLight Analytics, Nova Scotia, Canada). Each tooth sample was positioned above the input sensor of the spectrophotometer, and the transmitted radiant emittance (mW/cm2) was recorded during each step of the different restorative procedures with a model curing light emitting either blue (460nm), green (520nm), or red (620nm) at 1000mw/cm2. The adhesive system was light-cured for 10 seconds. The conventional composite was cured for 20 seconds per increment (two increments). The bulk-fill composite and the resin cement were each light-cured for 40 seconds. - Thermal analysis The prepared teeth were placed in a customized oral cavity chamber simulator in which the temperature was maintained at 37oC (±2oC) using a digitally controlled circulating water bath. Each tooth was secured to the chamber simulator with the pulp chamber oriented toward an infrared camera (FLIR ONE PRO, FLIR Systems) with thermal sensitivity of &lt;0.10oC and 150 mK to continuously record temperature variation (oC) during the light-activation procedures described above. Each specimen was allowed to equilibrate in the chamber at 37°C for 10 minutes prior to recording. Because infrared thermography is sensitive to emissivity and environmental conditions, temperature outcomes were analyzed and reported primarily as T (°C), defined as the change from baseline recorded immediately before irradiation for each specimen, rather than as absolute temperature values. Additionally, to improve measurement consistency, temperature was recorded only at the center of the pulpal chamber for all specimens. All measurements were performed under consistent camera settings and environmental conditions to minimize variability related to emissivity and reflection. Thermal videos were exported using FLIR Tools and converted to a data sheet for analysis. - Cell viability analysis Transmitted radiant emittance measured at the pulpal floor during each restorative procedure was used to calculate the radiant exposure (J/cm2) that could reach pulp-side cells under the different scenarios tested. These calculated exposures were used to define the worst-case bounding dose approach for the cell viability analysis described below. Immortalized odontoblastic-like cells (MDPC-23) were cultivated in Dulbecco's Modified Eagle's Medium (DMEM) (Sigma Aldrich, St. Louis, MO, USA) supplemented with 10% fetal bovine serum (FBS) (Sigma Aldrich, St. Louis, MO, USA). The medium was then replaced with DMEM containing only 0.5% FBS to reduce growth-factor availability and approximate a metabolically stressed condition. Cells were irradiated with blue (460 nm), green (520 nm), or red (620 nm) light using the maximum radiant exposure measured at the pulpal floor across the tested restorative scenarios (17 J/cm²; worst-case exposure) (n = 5). Non-irradiated cells were used as controls. Cell viability was assessed using an MTT assay (MTT Assay, MTT Assays Life Technologies Corp., Eugene, OR, USA) according to the manufacturer's instructions. Briefly, after incubation, the supernatant in each well was carefully removed and replaced with 100 µL of absolute alcohol (Sigma Aldrich, St. Louis, MO, USA) to dissolve the formazan crystals. The optical density of the solution was measured at 570 nm using a 96-well plate absorbance reader (Byonoy, Hamburg, Germany). Experiments were performed in triplicate, and the mean of the fifteen readings was used for statistical analysis. - Statistical Analysis A priori power analysis was performed to determine the minimum sample size required to achieve 80% power at = 0.05. Specimens were randomized based on baseline light-transmittance values to distribute samples with comparable optical characteristics across wavelength groups. Normality and homogeneity of variances were evaluated using the Shapiro-Wilk and Levene's tests, respectively. For each restorative procedure and curing step, differences among wavelengths were analyzed using one-way ANOVA followed by Tukey's post hoc test ( = 0.05). Cell-viability (MTT) data were analyzed separately; percent viability was calculated relative to the non-irradiated control, and group differences (blue, green, and red) were evaluated using one-way ANOVA with Tukey's post hoc test ( = 0.05).

## Results

- Light Transmittance and Radiant Exposure Analyses Tables 2 and 3 show the light transmittance (mW/cm2) and the radiant exposure (J/cm2) of the different wavelengths reaching the pulpal floor during the different restorative procedures evaluated: conventional composite restoration, bulkfill composite restoration, and indirect ceramic cementation.

Table 2

Table 3

Across all restorative procedures evaluated, an overall trend was observed in which light transmittance and radiant exposure increased with longer wavelengths (p &lt; 0.05). Specifically, red light (longest wavelength) consistently showed the highest irradiance through the pulp floor, followed by green and then blue light, regardless of the restorative technique. Additionally, increased material thickness led to a systematic reduction in light transmittance and radiant exposure for all wavelengths (p &lt; 0.001). Whether due to additional composite layers, bulk-fill increments, or the presence of ceramic restorations and resin cement, thicker or more optically dense structures progressively attenuated light transmission. This attenuation was observed consistently across all three light sources, though the degree of reduction varied with wavelength: longer wavelengths (red) were less affected than shorter ones (blue). - Thermal Analysis Table 4 shows the temperature increase (°C) generated by the different wavelengths during the different restorative procedures evaluated.

Table 4

**Figure 2:**
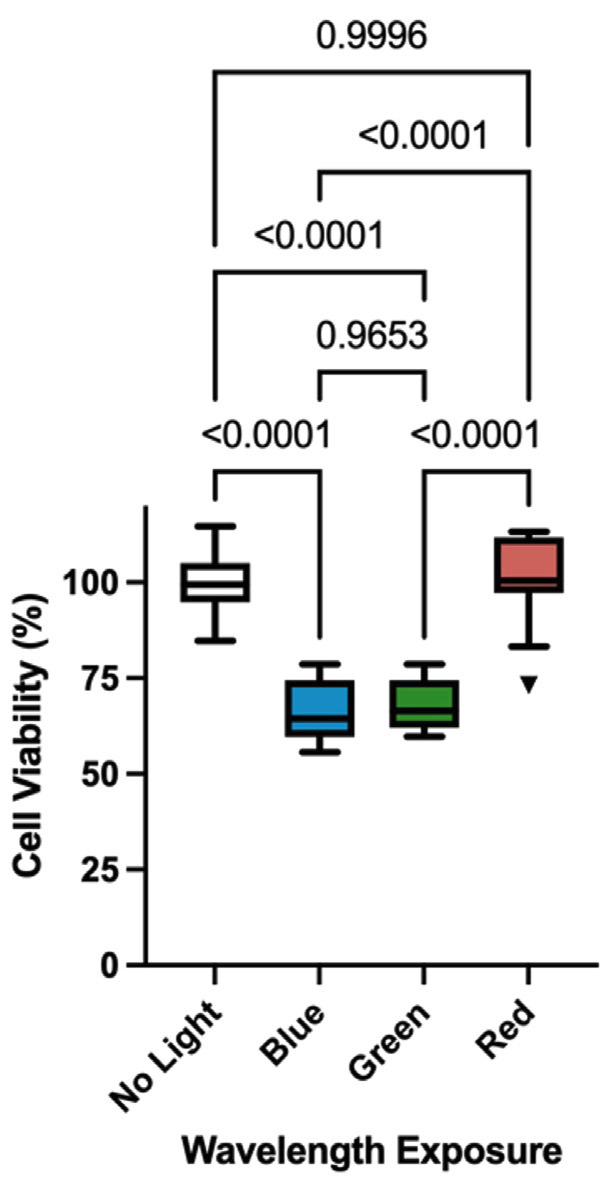
2

Across all procedures, a general trend was observed in which temperature rise was greater at shorter wavelengths, with blue light leading to the highest temperature increases, particularly during the curing of thicker composite increments (p &lt; 0.05). In contrast, red light consistently produced the lowest temperature increases, with values significantly lower than both green and blue at most steps of the composite procedure (p &lt; 0.01). The temperature rise also increased systematically with curing time, especially under blue and green light (p &lt; 0.001), while red light showed a more stable thermal profile with smaller variations between procedural steps. - Cell Viability Analysis Figure 2 shows the cell viability after irradiation with the different wavelengths with the maximum radiant exposure that reached the pulpal chamber according to the different restorative scenarios tested.

2

Exposure to blue and green light significantly reduced cell viability compared to the non-irradiated control group (p &lt; 0.05). In contrast, cells exposed to red light maintained viability levels statistically similar to the control group (p = 0.23), suggesting that red light did not adversely affect cell metabolism under the tested conditions.

## Discussion

As observed in our results, longer wavelengths exhibited significantly higher light transmittance across all tested restorative procedures, including conventional composites, bulk-fill composites, and ceramic restorations. These findings supported our first hypothesis that long wavelengths would result in greater light transmittance through different restorative materials compared to shorter wavelengths. Shorter wavelengths, such as blue and violet light, have limited ability to be transmitted through resin-based materials (6 , 13 , 14). This happens due to a phenomenon called Rayleigh Scattering. Rayleigh scattering occurs when light interacts with particles smaller than its wavelength. Shorter wavelengths are closer in size to these particles, leading to greater scattering than longer wavelengths (8) As scattering increases, light is attenuated in depth (6), reducing the amount of energy that reaches the deeper layers of the restoration. The insufficient energy prevents the photoinitiators from sustaining polymerization at greater depths (15). In contrast, longer wavelengths, such as red light, experience less scattering and allow greater light transmission, supporting depth of cure.6,8 This has important clinical implications, particularly in bulk fill restorations, the proximal boxes of Class II restorations, and indirect cementation procedures, where resin materials may need to be cured through thicknesses exceeding 4 millimeters (15). Our results also showed that red light caused a lower increase in temperature within the resin-based materials than blue or green light. In addition, cell viability was highest following exposure to red light compared to other wavelengths. Therefore, our second proposed hypothesis that long wavelengths would generate less heat and induce less cell damage compared to short wavelengths was also accepted. In fact, shorter wavelengths, such as blue light, are more energetic and carry higher photon energy than longer wavelengths at equivalent radiant emittances (6 - 8). Photon energy not absorbed by the photoinitiators is released as heat into the surrounding tissues, potentially causing harmful effects (7 , 9). In contrast, longer wavelengths carry lower photon energy, resulting in less excess energy being dissipated as heat to the surrounding tissues (7). Heat-induced stress can disrupt cellular metabolic processes and compromise protein stability, leading to impaired cellular function and reduced viability, as observed in this study. Specifically, excess heat can denature proteins, alter enzyme activity, and damage cellular membrane integrity (9 , 10). In our in vitro conditions, blue light produced the greatest increase in temperature across all restorative processes evaluated and resulted in the lowest cell viability compared to the longer wavelengths. These findings suggest a wavelength-dependent biological burden under controlled exposure conditions, but they should not be interpreted as direct evidence of clinical pulpal injury. Instead, as a demonstration of the potential benefits of longer wavelengths in Dentistry. As an example, red light, has been reported as an adjunct modality in photobiomodulation for conditions such as mucositis, dentinal hypersensitivity, and pulpal stimulation, and has been investigated for effects on pulpal cells (16). In vitro studies suggest that red light may enhance cell metabolism, as long wavelengths can be absorbed by cytochrome c oxidase, potentially increasing adenosine triphosphate (ATP) synthesis and cellular energy availability (16). Red light also stimulates the synthesis of collagen-rich matrix and essential proteins involved in tissue repair and regeneration (10 , 16). Additionally, red light has been linked to the formation of mineralized tissue and tertiary dentin deposition in some experimental models (16). Together, these cellular responses may support repair of the pulp-dentin complex following injury (16). In the context of restorative dentistry, these reports provide a biologic rationale for further investigation. Based on our findings, we observed wavelength-dependent differences in transmission and thermal response (6 , 13) and noted that red light offers several benefits, including greater light transmittance, lower heat generation, and the potential for cellular photobiomodulation (10 , 16). A limitation of our study is that all our results were obtained in a simulated, in vitro environment, which does not fully replicate physiological conditions. The model does not reproduce pulpal blood flow-mediated heat dissipation, interpatient variability in remaining dentin thickness and dentin optical properties, or patientrelated conditions that may modify thermal and biological responses. Moreover, the study design represents a simplified, worst-case scenario in vitro model. Therefore, we cannot say with certainty that these same results would directly translate to different clinical scenarios. In fact, previous research has shown that in vitro models tend to overestimate temperature increases in pulpal tissues compared to in vivo conditions (17). To validate the clinical relevance of our findings, future in vivo studies are necessary, and our conclusions should be viewed as hypothesis-generating. Additionally, although red-light photobiomodulation effects have been described under specific dose conditions, the present study did not evaluate photobiomodulation endpoints (e.g., proliferation, differentiation markers, inflammatory mediators); therefore, our findings should be interpreted as indicating lower cytotoxicity under the tested exposure conditions, not as evidence of a photobiomodulation therapeutic effect. Future studies using physiologic dentin-pulp models and clinically representative exposure conditions are needed to determine whether the observed wavelength-dependent effects persist in vivo and to define their clinical relevance. It is important to emphasize that these observations do not directly establish clinical risk associated with contemporary light-curing units. The relationship between light-curing exposure and pulpal injury in vivo remains uncertain (17). Clinical pulpal complications are more commonly associated with diagnostic and restorative factors rather than photoactivation alone. The present study instead provides laboratory data describing how wavelength influences transmission and pulpal cell response when radiant emittance is held constant. The model used in this study requires careful interpretation. First, the remaining dentin thickness was standardized at 0.5 mm, representing a worst-case scenario that likely overestimates pulpal exposure relative to most clinical situations, and does not capture the variability in remaining dentin among patients and preparations. Second, pulp-chamber temperature measurements were obtained using infrared thermography, which can be influenced by emissivity assumptions, reflected background temperature, and environmental conditions; therefore, temperature outcomes should be interpreted primarily as relative T trends rather than exact absolute pulp temperatures. Third, the biological assays used an odontoblast-like cell line and in vitro exposure conditions that cannot fully replicate pulp physiology; thus, the biological effects observed cannot be extrapolated directly to clinical outcomes. Although we assessed polymerization performance (degree of conversion) for the experimental materials evaluated in this study, these results are specific to the tested multi-wavelength photoinitiator system. Therefore, the wavelength-dependent findings on light transmission, heat generation, and cellular response should not be directly extrapolated to conventional camphorquinone- or TPO-based commercial composites without confirming comparable performance under the same irradiation conditions - but a proof of concept. While similar trends may be observed when these limitations are addressed, the magnitude of the effects may differ under clinically relevant conditions and curing protocols. For this reason, further work is necessary to validate the clinical relevance of these findings in vivo. Still, despite these limitations, the present data indicate that, when radiant emittance was held constant, longer wavelengths, particularly red, were associated with higher transmittance to the pulpal floor, lower temperature rise, and preserved cell viability under the tested conditions.

## Conclusions

Within the limitations of this in vitro model, it was possible to conclude that longer wavelengths demonstrate increased light transmission, lower temperature rise, and higher cell viability than shorter wavelengths delivered at equal exposure. These preliminary findings describe wavelength-dependent cellular response under controlled conditions, supporting further investigation.

## Figures and Tables

**Table 1:** Monomers and fillers used in the formulation of the experimental resin-based materials tested.

Material	Chemical	Manufacturer
Monomers	Bis-GMA*	Sigma Aldrich, St. Louis, MO, USA
	Bis-EMA*	
	UDMA*	
	TEGDMA*	
	HEMA*	
Fillers	ExothaneTM 24	Esstech Inc., Essington, PA, USA
	0.05 Î¼m fumed silica	Nippon Aerosil Co. Ltd., Tokyo, Japan
	0.7 Î¼m BaBSiO2*	Esstech Inc., Essington, PA, USA

* Bisphenol A diglycidylmethacrylate (Bis-GMA); Ethoxylated bisphenol A diglycidyldimethacrylate (Bis-EMA); Triethylene glycol dimethacrylate (TEGDMA); (Hydroxymethyl)methacrylate (HEMA); Urethane dimethacrylate (UDMA); Barium borosilicate glass (BaBSiO2).

**Table 2:** Light transmittance (mW/cm2) of different wavelength spectra through different restorative procedures.

Material	Spectra	Light-Transmittance (mW/cm2)											
Composite		Initial			Adhesive (10s)			Increment 1 (20s)			Increment 2 (20s)		
	Blue	243	(62.4)	C	257	(75.6)	C	65	(8.0)	C	29	(2.0)	C
	Green	387	(16.0)	B	411	(15.9)	B	79	(22.5)	B	31	(11.9)	B
	Red	405	(30.8)	A	441	(27.8)	A	189	(25.5)	A	106	(18.2)	A
Bulkfill		Initial			Adhesive (10s)			Single Increment (40s)					
	Blue	264	(30.0)	C	280	(35.2)	C	24	(6.1)	C			
	Green	384	(19.6)	B	406	(14.0)	B	31	(9.2)	B			
	Red	478	(11.8)	A	437	(12.7)	A	97	(6.6)	A			
Ceramic		Initial			Adhesive (10s)			Resin Cement (40s)					
	Blue	331	(66.1)	C	339	(62.6)	C	129	(20.2)	C			
	Green	565	(44.6)	B	562	(50.5)	B	220	(10.6)	B			
	Red	580	(84.0)	A	583	(78.4)	A	267	(23.5)	A			

* Different letters indicate significant differences between wavelengths in each step (p < 0.05).

**Table 3:** Radiant Exposure (J/cm2) from different wavelength spectra reaching the pulpal floor in different restorative procedures.

Material	Spectra	Radiant Exposure (J/cm2)											
Composite		Adhesive (10s)			Increment 1 (20s)			Increment 2 (20s)			Total		
	Blue	2.63	(0.6)	C	1.31	(0.1)	C	0.57	(0.1)	C	4.51	(0.8)	C
	Green	4.27	(0.2)	B	1.59	(0.4)	B	0.64	(0.2)	B	6.50	(0.8)	B
	Red	4.57	(0.4)	A	3.84	(0.5)	A	2.16	(0.4)	A	10.52	(1.2)	A
Bulkfill		Adhesive (10s)			Single Increment (40s)			Total					
	Blue	2.91	(0.3)	C	3.88	(0.2)	C	6.79	(0.6)	C			
	Green	4.16	(0.1)	B	5.58	(0.3)	B	9.74	(0.3)	B			
	Red	4.42	(0.1)	A	8.38	(0.3)	A	12.80	(0.1)	A			
Ceramic		Adhesive (10s)			Resin Cement (40s)			Total					
	Blue	3.51	(0.7)	C	5.20	(0.8)	C	8.71	(1.5)	C			
	Green	5.78	(0.5)	B	8.93	(0.4)	B	14.71	(1.0)	B			
	Red	5.97	(0.9)	A	10.83	(0.9)	A	16.80	(1.8)	A			

* Different letters indicate significant differences between wavelengths in each step or overall accumulation (p < 0.05).

**Table 4:** Temperature variation (ΔT, oC) at the pulpal floor during exposure to different wavelength spectra across different restorative procedures.

Material	Spectra	Δ Temperature (oC)								
Composite		Adhesive (10s)			Increment 1 (20s)			Increment 2 (20s)		
	Blue	5.97	(3.4)	A	20.17	(2.9	A	5.93	(1.8)	A
	Green	3.27	(1.9)	B	11.31	(1.5)	B	5.71	(1.7)	B
	Red	2.17	(3.2)	C	3.65	(3.1)	C	3.58	(2.9)	C
Bulkfill		Adhesive (10s)			Single Increment (40s)					
	Blue	7.61	(1.6)	A	19.41	(1.9)	A			
	Green	3.27	(1.1)	B	9.44	(2.1)	B			
	Red	2.39	(1.6)	C	7.48	(1.4)	C			
Ceramic		Adhesive (10s)			Resin Cement (40s)					
	Blue	9.85	(1.2)	A	8.64	(0.9)	A			
	Green	2.73	(1.5)	B	3.90	(1.5)	B			
	Red	0.95	(1.8)	C	2.98	(1.4)	C			

* Different letters indicate significant differences between wavelengths in each step (p < 0.05).

## Data Availability

The datasets used and/or analyzed during the current study are available from the corresponding author.
